# Association of *FOXP3* Gene Polymorphisms with Skin, Vulvar and Ectocervical Squamous Cell Tumors

**DOI:** 10.3390/medicina62102000

**Published:** 2026-10-15

**Authors:** Boba Kotlica, Katarina Stefanović, Giuseppe Argenziano, Milan Stojičić, Goran Stojković, Sveto Pantović, Lidija Tulić, Anđa Ćirković, Anđela Dimkić Milenković, Nikola Todorović, Ivana Novaković

**Affiliations:** 1 Faculty of Medicine, University of Belgrade, 11000 Belgrade, Serbia; jeremick@hotmail.com (K.S.); doktorat123@gmail.com (M.S.); drgoranstojkovic@gmail.com (G.S.); pant65@ymail.com (S.P.); coccolo13@yahoo.com (L.T.); 2 Clinic for Gynecology and Obstetrics, University Clinical Center of Serbia, 11000 Belgrade, Serbia; 3 Dermatology Unit, University of Campania “Luigi Vanvitelli”, 81100 Naples, Italy; g.argenziano@gmail.com; 4 Clinic for Burns, Plastic and Reconstructive Surgery, University Clinical Center of Serbia, 11000 Belgrade, Serbia; adimkic@yahoo.com; 5 Clinic for Otorhinolaryngology and Maxillofacial Surgery, University Clinical Center Serbia, 11000 Belgrade, Serbia; nikola.todorovic89@hotmail.com; 6 Institute for Medical Statistics and Informatics, Faculty of Medicine, University of Belgrade, 11000 Belgrade, Serbia; 7 Institute of Human Genetics, Faculty of Medicine, University of Belgrade, 11000 Belgrade, Serbia; ivana.novakovic@med.bg.ac.rs

**Keywords:** *FOXP3*, rs2280883, rs3761548, actinic keratosis, Bowen’s disease, VIN, LSIL, HSIL, SCC

## Abstract

*Background and Objectives*: Squamous cell tumors arise from squamous epithelial cells. Polymorphisms in the *FOXP3* gene may influence malignancy susceptibility. This study evaluated the potential association of *FOXP3* rs3761548 (−3279 A/C) and rs2280883 (IVS9+459 T/C) polymorphisms with preinvasive and invasive squamous cell tumors of sun-exposed skin, sun-protected vulvar skin, and the ectocervix. *Materials and Methods*: This comparative observational study included 135 women aged >18 years surgically treated for squamous cell tumors of the skin (actinic keratosis, Bowen’s disease, squamous cell carcinoma), vulva (HSIL, differentiated VIN, squamous cell carcinoma), or ectocervix (LSIL, HSIL, squamous cell carcinoma). Sociodemographic, anthropometric, lifestyle, ultraviolet radiation exposure, reproductive, and sexual health data were collected. The *FOXP3* rs3761548 and rs2280883 polymorphisms were genotyped; codominant, dominant, recessive, and overdominant genetic models were evaluated. *Results*: The three groups differed significantly in several sociodemographic, anthropometric, lifestyle, reproductive, and sexual health characteristics. No significant differences were observed for smoking, sun exposure, history of sunburns, contraceptive use, or history of abortions. The minor A allele frequency of rs3761548 was lower in the skin and vulvar tumor groups and slightly higher in the ectocervical tumor group than in reference populations; however, this difference was not significant (*p* = 0.066). None of the rs3761548 genetic models remained significant after LASSO regression. For rs2280883, the C allele frequency was lower in the skin and ectocervical groups but higher in the vulvar tumor group than the 46.0% reported in the reference population. The dominant model (CC + CT vs. TT) was associated with lower odds of skin tumors, whereas the recessive model (CC vs. TT + CT) was associated with higher odds (both *p* = 0.037). *Conclusions*: The distribution of the *FOXP3* rs2280883 polymorphism differed according to tumor location. It was significantly associated with cutaneous squamous cell tumors under both dominant and recessive genetic models.

## 1. Introduction

Squamous cell tumors are neoplasms arising from squamous epithelial cells, which constitute the surface layer of the skin (epidermis) and line various mucosal surfaces. In addition to the skin, squamous cell carcinomas (SCCs) may develop in the lungs and bronchi, oral cavity, anogenital region, and gastrointestinal tract. Generally speaking, preinvasive lesions precede the development of cancer. When a neoplastic lesion extends through the full thickness of the epithelium and breaches the basement membrane, it is classified as invasive SCC. They have the potential to metastasize via the lymphatic and hematogenous routes [1].

SCC of chronically sun-exposed skin is an invasive tumor that accounts for approximately 20–50% of all skin cancers, with the number of newly diagnosed cases continuously increasing. Although the mortality rate associated with this carcinoma is low, its high incidence and the procedures required for treatment impose a significant economic burden on both patients and the healthcare system [2]. Ultraviolet radiation (UV) with a wavelength in the range of 280–320 nanometers affecting chronically exposed regions, such as the face, bald scalp, or forearms, has been found to cause DNA mutations in keratinocytes. If the mutations are irreparable, atypical cells begin to grow uncontrollably beyond basement membrane. The penetration of malignant cells into the dermis results in the development of an invasive tumor, namely, SCC. This process can be accelerated if the patient is receiving immunosuppressive therapy or has a human papillomavirus (HPV) infection. Other significant factors contributing to the development of skin SCC include ionizing radiation, heat, chronic inflammation, petroleum products, tobacco and tar [3]. The tumor exhibits rapid exophytic growth, followed by ulceration with bleeding and the potential for metastasis.

The most common preinvasive skin tumors are actinic keratosis (AK) and Bowen’s disease. It is believed that almost every White person with skin phototypes I and II develops at least one AK during their lifetime, and often several. AK manifests as a reddish, rough, and painless lesion. If left untreated, 5% of cases progress to an invasive tumor. Bowen’s disease can be caused by ultraviolet radiation, HPV infection, or exposure to arsenic. It presents as a slowly growing plaque that can occur anywhere on the body and is sometimes associated with mild pruritus. The appearance of ulceration indicates progression to an invasive squamous cell tumor (Bowen’s carcinoma) [4].

In sun-protected skin, invasive SCC is the most common malignant tumor, particularly of the vulva. It is a disease that primarily affects older women in the eighth decade of life, but it can also occur during the perimenopausal period. Most patients are smokers and have a history of HPV infection. Pruritus and a sensation of irritation, as well as detection of a rough surface during personal hygiene, are often overlooked as symptoms of the disease, resulting in delayed treatment [5].

Vulvar intraepithelial neoplasia (VIN) is a preinvasive squamous cell tumor of the vulvar skin that may represent a precursor to a serious malignant disease. The mildest form of squamous atypia, limited to the lower part of the epidermis, is classified as low-grade squamous intraepithelial lesion (LSIL) and may develop as reactive atypia or as a result of HPV infection, clinically manifested by the appearance of condylomas. High-grade squamous intraepithelial lesion (HSIL), or VIN usual type, previously referred to as VIN 2 and VIN 3, is strongly associated with HPV infection. Clinically, these lesions appear as whitish, reddish, or darkly pigmented lesions of varying sizes that can occur anywhere on the vulva. According to the International Society for the Study of Vulvovaginal Disease, a special form of VIN 3, formerly referred to as the simplex type, is now classified as differentiated type VIN (DVIN) [6]. Clinically, it manifests as a hyperkeratotic plaque and is associated with dermatoses, such as lichen sclerosus and lichen planus, rather than HPV infection. The tumor progresses rapidly toward the development of invasive SCC of the vulva [7].

SCC of the uterine cervix, i.e., the ectocervix, is an invasive tumor and the fourth most common malignancy among women worldwide, with approximately 600,000 newly diagnosed cases per year. In Europe, this tumor affects approximately 58,000 women (10.7 per 100,000), while 26,000 women (3.76 per 100,000) die annually [8,9]. However, the mortality rate from cervical cancer is decreasing, mainly due to cytological screening, HPV testing, and the use of HPV vaccines. Preinvasive tumors of the ectocervix include LSIL and HSIL. Several risk factors are associated with the development of cervical tumors, including HPV infection, particularly infection with highly oncogenic HPV types, immunosuppression, human immunodeficiency virus infection, smoking, and a higher number of sexual partners. The symptoms and signs of early-stage cervical cancer are often subtle and may include vaginal discharge, occasional spotting, or vaginal bleeding after intercourse. These findings highlight the importance of regular gynecological examinations, which enable early detection and increase the likelihood of successful treatment.

Forkhead box P3 transcription factor (*FOXP3*) is a member of the forkhead family of transcription factors. Through its interaction with DNA, *FOXP3* can induce either chromatin compaction or decondensation, thereby restricting or permitting access to other transcriptional regulatory proteins [10,11]. As a marker of regulatory T lymphocyte identity, *FOXP3* plays a crucial role in maintaining immune tolerance, regulating immune responses, and promoting the development and function of regulatory T cells (Tregs) [12].

Alterations in *FOXP3* expression can lead to abnormal production of Tregs, while reduced *FOXP3* expression may contribute to the development of certain types of cancer in humans [13]. *FOXP3* expression contributes to the maintenance of an immunosuppressive state that supports HPV infection and the development of squamous intraepithelial tumors [14]. Previously, *FOXP3* was thought to be expressed exclusively in Tregs. However, subsequent studies have demonstrated that *FOXP3* is also expressed in tumor cells, although its effects appear to be contradictory. Furthermore, increased *FOXP3* expression has been observed in tumor cells themselves, which may, in practical terms, be used to evaluate the effects of certain drugs [15].

*FOXP3* is encoded by a gene located on the X chromosome at the Xp11.23 region [14]. The 2025 Nobel Prize laureates in Physiology or Medicine, Mary E. Brunkow and Fred Ramsdell, demonstrated that mutations in the *FOXP3* gene cause IPEX syndrome in humans, a severe autoimmune disorder [16]. Subsequently, Shimon Sakaguchi established the critical role of *FOXP3* in the development and function of Tregs [12].

Multiple polymorphisms have been identified within the *FOXP3* gene sequence, representing normal variation in the genetic material. These are predominantly single-nucleotide polymorphisms (SNPs). The polymorphic loci rs3761548 (−3279 A/C) and rs2280883 (IVS9+459 T/C) are located in regulatory regions of the *FOXP3* gene [11]. The rs3761548 (−3279 A/C) polymorphism is located in the promoter, whereas rs2280883 (IVS9+459 T/C) polymorphism is located in the intron 9 region. These polymorphisms affect the level of transcription, meaning that certain alleles and genotypes are associated with increased or decreased gene expression and protein synthesis [11,17]. Numerous association studies have analyzed these polymorphisms as potential predisposing factors for the development of various diseases, including malignancies [17,18,19].

However, relatively few studies have investigated *FOXP3* expression or functional *FOXP3* polymorphisms in cervical or cutaneous squamous cell tumors [20,21].

Based on these considerations, our study aimed to evaluate the potential association between the *FOXP3* genetic variants rs3761548 (−3279 A/C) and rs2280883 (IVS9+459 T/C) and different locations of preinvasive and invasive squamous cell tumors arising on sun-exposed skin, sun-protected vulvar skin, and the ectocervix.

## 2. Materials and Methods

### 2.1. Study Design and Patient Selection

The study was designed as an observational study comparing different case groups. A total of 135 female patients aged >18 years who underwent surgical treatment for squamous cell tumors involving the following anatomical sites were included: sun-exposed skin (preinvasive including AK and Bowen’s disease, and invasive SCC); sun-protected skin, specifically the vulva (preinvasive HSIL and DVIN and invasive SCC); and the ectocervix (preinvasive LSIL and HSIL, and invasive SCC).

Female patients were treated between June 2025 and June 2026 at the Clinic for Gynecology and Obstetrics, University Clinical Center of Serbia (UCCS); the Clinic for Burns, Plastic and Reconstructive Surgery, UCCS; the Clinic for Otorhinolaryngology and Maxillofacial Surgery, UCCS; and the Clinic for Gynecology and Obstetrics “Narodni front.” Blood samples were collected from all participating patients, transported to the Institute of Human Genetics, Faculty of Medicine, University of Belgrade (FMUB), and subsequently analyzed.

All participants provided written informed consent for inclusion in the study. All patient data were collected and processed in accordance with the principles of the Declaration of Helsinki, with strict adherence to patient confidentiality and anonymity. Ethical approval was obtained from the Ethics Committee of the UCCS (Decision No. 1128/23, 3 July 2025; Amendment No. 1500/48, 29 September 2025), the Ethics Committee of the Clinic for Gynecology and Obstetrics “Narodni Front” (Decision No. 2208/2025/021590, 9 October 2025), and the Institute of Human Genetics, FMUB (Decision No. 108, 5 June 2025).

The inclusion criteria were a biopsy-confirmed tumor, a dermoscopically suspicious tumor of sun-exposed and sun-protected (vulvar) skin or persistent abnormal cervical cytology test. Subjects whose histopathological findings did not confirm the presence of preinvasive or invasive SCC were excluded from the study.

### 2.2. Data Collection

As part of the study, data were collected on the participants’ sociodemographic characteristics, anthropometric parameter, lifestyle habits, and history related to UV exposure. Sociodemographic data included age, educational level, marital status, and employment status. Body mass index (BMI) was assessed as an anthropometric parameter. Lifestyle-related data included cigarette smoking and alcohol consumption. Assessment on UV radiation exposure included sun exposure, tanning bed use, and the occurrence of sunburns. Additionally, data on reproductive and sexual health characteristics were collected. These included age at menarche, age at first sexual intercourse, number of sexual partners during lifetime, number of sexual partners during the previous six months, contraceptive use, history of sexually transmitted infections, parity, and previous abortions.

### 2.3. FOXP3 Polymorphism Genotyping

For the analysis of *FOXP3* gene polymorphisms, 10 mL of whole blood collected in sodium citrate tubes was obtained preoperatively from all subjects by peripheral venipuncture and transported to the laboratory of the Institute of Human Genetics, FMUB. DNA extraction was performed using the salting-out method [22].

Genotyping of the *FOXP3* rs3761548 and rs2280883 polymorphisms was performed using pre-designed TaqMan SNP assays (Thermo Fisher Scientific Inc., Waltham, MA, USA) on an Applied Biosystems 7500 Real-Time PCR System (Thermo Fisher Scientific Inc., Waltham, MA, USA).

### 2.4. Statistical Analysis

Categorical data were presented as absolute frequencies and percentages. Differences in the distribution of categorical variables between independent groups were assessed using the chi-square test or Fisher’s exact test, as appropriate.

Four genetic models were assessed for each *FOXP3* SNP: codominant, dominant, recessive, and overdominant. For rs3761548, the codominant model was defined as CC vs. AC vs. AA, the dominant model as AA + AC vs. CC, the recessive model as AA vs. AC + CC, and the overdominant model as AC vs. AA + CC. For rs2280883, the corresponding models were CC vs. CT vs. TT, CC + CT vs. TT, CC vs. TT + CT, and CT vs. TT + CC, respectively [14].

Hardy–Weinberg equilibrium (HWE) was assessed using the chi-square test in each study group. Associations between genetic models and the presence of skin, vulvar, and ectocervical tumors were evaluated using univariable logistic regression. Subsequently, least absolute shrinkage and selection operator (LASSO) binary logistic regression was performed using the glmnet package in R to identify the most relevant predictors of the binary outcome. The LASSO model was fitted using a binomial distribution with a logit link and an L1 penalty (α = 1). The optimal penalty parameter (λ) was selected using 10-fold cross-validation. Two λ values were considered: λ_min, corresponding to the minimum cross-validated error, and λ_1se, defined as the largest λ value within one standard error of the minimum cross-validated error. The λ_1se criterion was used for the final model to obtain a more parsimonious set of predictors. Regression coefficients were extracted at λ_1se, and their exponentiated values (exp[β]) were reported as penalized odds ratios (ORs).

A *p*-value of < 0.05 was considered statistically significant. Statistical analyses were performed using IBM SPSS Statistics for Windows, Version 31 (IBM Corp., Armonk, NY, USA).

## 3. Results

### 3.1. Characteristics of the Study Participants

The study included 135 women with squamous cell tumors of the skin (49/135), vulva (33/135), and ectocervix (53/135), with a median age of 62 years (range, 20–95 years). The sociodemographic data, anthropometric parameter, lifestyle habits, factors related to UV radiation exposure, and reproductive and sexual health characteristics of the study participants are presented in Table 1.

The three study groups differed significantly in the distribution of age, anthropometric parameter, education level, marital status, employment status, alcohol consumption, tanning bed use, age at menarche, age at first sexual intercourse, lifetime number of sexual partners, having a sexual partner during the previous 6 months, history of sexually transmitted infection, and parity (*p* < 0.001, *p* < 0.001, *p* < 0.001, *p* < 0.001, *p* < 0.001, *p* < 0.001, *p* = 0.004, *p* = 0.040, *p* = 0.001, *p* < 0.001, *p* = 0.002, and *p* = 0.002, respectively). However, no significant differences were observed between the groups in terms of smoking status, sun exposure, history of sunburns, contraceptive use, or history of abortions (all *p* > 0.05). Women with skin and vulvar tumors were significantly older than those with ectocervical tumors. Specifically, 92% of women with skin tumors and 79% of those with vulvar tumors were aged ≥55 years, compared with only 13% of women with ectocervical tumors (*p* < 0.001 for both comparisons). Similarly, lower educational attainment (primary or no education) and unemployment were more common among women with skin and vulvar tumors than among those with ectocervical tumors. Primary or no education was reported in 25% of women with skin tumors and 30% of those with vulvar tumors, compared with none of the women with ectocervical tumors (*p* = 0.001 and *p* < 0.001, respectively). Unemployment was more frequently reported among women with skin and vulvar tumors (84% and 82%, respectively) than among those with ectocervical tumors (25%), with both comparisons reaching statistical significance (*p* < 0.001). In contrast, the proportion of single women was significantly lower in these two groups than among women with ectocervical tumors (*p* < 0.001 and *p* = 0.007, respectively). Overall, alcohol consumption was most frequent among women with ectocervical tumors, reported in 72% of cases. Alcohol consumption differed significantly among the three groups, being more common among women with ectocervical tumors than among those with skin tumors, and among those with vulvar tumors (*p* = 0.032 and *p* < 0.001, respectively). A significant difference was also observed between the skin and vulvar tumor groups (*p* = 0.032). Tanning bed use was significantly more common among women with ectocervical tumors (60.4%) than among those with skin (8.2%; *p* < 0.001) or vulvar tumors (12.1%; *p* < 0.001). Overweight women were more common in the skin tumor group than in the ectocervical tumor group (*p* = 0.022), while obese women were more common in the vulvar tumor group than in the ectocervical tumor group (*p* = 0.016). Regarding sexual lifestyle, gynecological, and obstetric characteristics, women with ectocervical tumors more frequently reported menarche before 13 years of age (42%), first sexual intercourse at ≤17 years of age (47%), four or more lifetime sexual partners (64%), and one or more sexual partners during the preceding 6 months (85%) compared with women in the other tumor groups. The history of sexually transmitted infections was also most frequently reported in the ectocervical tumor group (91%). Parity differed significantly among the groups, with nulliparity being more common among women with ectocervical tumors than among those with skin tumors (32% vs. 8%, *p* < 0.001), and among women with vulvar tumors than among those with skin tumors (18% vs. 8%, *p* = 0.043).

### 3.2. FOXP3 Genetic Variants and Association with Skin, Vulvar, and Ectocervical Tumors

The distribution of *FOXP3* genotypes and alleles according to tumor location is presented in Table 2. For rs3761548, the observed genotype frequencies in the skin tumor group were 25/49 (51.0%), 13/49 (26.5%), and 11/49 (22.5%) for CC, AC, and AA genotypes, respectively. In the vulvar tumor group, the genotype frequencies were 11/33 (33.3%), 14/33 (42.4%), and 8/33 (24.2%) for CC, AC, and AA genotypes, respectively. In the ectocervical tumor group, the genotype frequencies were 12/53 (22.6%), 27/53 (50.9%), and 14/53 (26.4%) for CC, AC, and AA genotypes, respectively. The calculated allele frequencies were 35.7% (A) and 64.3% (C) in the skin tumor group; 45.4% (A) and 54.6% (C) in the vulvar tumor group; and 51.9% (A) and 48.1% (C) in the ectocervical tumor group. Compared with European reference populations, the minor allele frequency (MAF) of *FOXP3* rs3761548 (A, 47.8% in the 1000 Genomes European population), was lower in the skin and vulvar tumor groups but slightly higher in the ectocervical tumor group [23].

For rs2280883, the genotype frequencies in the skin tumor group were 9/49 (18.4%), 13/49 (26.5%), and 27/49 (55.1%) for CC, TC, and TT genotypes, respectively. In the vulvar tumor group, the corresponding frequencies were 6/33 (18.2%), 16/33 (48.5%), and 11/33 (33.3%), while in the ectocervical tumor group, they were 13/53 (24.5%), 26/53 (49.1%), and 14/53 (26.4%) for CC, TC, and TT genotypes, respectively. The corresponding allele frequencies were 31.6% (C) and 68.4% (T) in the skin tumor group, 37.9% (C) and 62.1% (T) in the vulvar tumor group, and 49.1% (C) and 50.9% (T) in the ectocervical tumor group. The C allele frequency was lower in the skin and ectocervical tumor groups and higher in the vulvar tumor group than the 46.0% reported in a UK Caucasian reference population [24].

The allelic distributions of the *FOXP3* polymorphisms rs3761548 and rs2280883 were subsequently compared among patients with skin, vulvar and ectocervical tumors. For rs3761548, the C allele was observed in 64%, 55%, and 48% of alleles from the skin, vulvar and ectocervical tumor groups, respectively, whereas the corresponding frequencies of the A allele were 36%, 45%, and 52%. No statistically significant differences in allelic frequencies were observed among the three groups (*p* = 0.066). Although an unadjusted pairwise comparison revealed a significant difference between the skin and ectocervical tumor groups (*p* = 0.020), this difference did not remain statistically significant after Bonferroni correction for multiple comparisons (adjusted *p* = 0.060). Similarly, no significant differences were observed between the skin and vulvar tumor groups (*p* = 0.211) or between the ectocervical and vulvar tumor groups (*p* = 0.412).

For rs2280883, the C allele frequencies were 32%, 38%, and 49% in the skin, vulvar, and ectocervical tumor groups, respectively, while the corresponding T allele frequencies were 68%, 62%, and 51%. A statistically significant difference in allelic distribution was observed among the three groups (*p* = 0.037). Pairwise analysis demonstrated a significant difference between the skin and ectocervical tumor groups (unadjusted *p* = 0.011), which remained statistically significant after Bonferroni correction (adjusted *p* = 0.033). In contrast, no significant differences were identified between the skin and vulvar tumor groups (*p* = 0.408) or between the ectocervical and vulvar tumor groups (*p* = 0.152).

HWE for rs3761548 and rs2280883 was assessed separately in the skin, vulvar, and ectocervical tumor groups. Genotype frequencies deviated significantly from HWE for both SNPs in the skin tumor group, whereas no significant deviations from HWE were observed in the ectocervical or vulvar tumor groups.

To evaluate the potential association between *FOXP3* SNPs and different tumor locations, the previously defined study groups were compared. Associations between genotype distributions and specific tumor locations were assessed using the codominant, dominant, recessive, and overdominant genetic models. For rs3761548, significant differences in genotype distribution among the tumor groups were observed under all genetic models except the recessive model, primarily reflecting differences between the skin and ectocervical tumor groups (*p* = 0.008, *p* = 0.003, and *p* = 0.012, respectively). For rs2280883, significant differences were observed under all genetic models, again primarily reflecting differences between the skin and ectocervical tumor groups (*p* = 0.011, *p* = 0.003, *p* = 0.003, and *p* = 0.019, respectively).

Following univariable logistic regression analysis of the *FOXP3* gene SNPs rs3761548 and rs2280883 in relation to the three tumor locations (Table 3), the codominant, dominant, and overdominant models for rs3761548 were found to be significantly associated with tumor location, primarily reflecting opposing associations between skin and ectocervical tumors. Under the codominant model, the AC genotype was associated with lower odds of skin tumor but higher odds of ectocervical tumor. Similarly, both the dominant and overdominant models were associated with lower odds of skin tumor and higher odds of ectocervical tumor. For rs2280883, the dominant, recessive, and overdominant models were significantly associated with skin tumors. The dominant and overdominant models showed protective associations, whereas the recessive model was associated with higher odds of skin tumors. In ectocervical tumors, the dominant model was associated with higher odds, while the recessive model was associated with lower odds. None of the evaluated genetic models for either of the two *FOXP3* SNPs showed a significant association with vulvar tumor group.

LASSO logistic regression (Table 4) was performed to identify factors associated with the presence of skin squamous cell tumors using codominant, dominant, recessive, and overdominant models of rs2280883, while also considering other clinically relevant characteristics, including sociodemographic data, anthropometric parameter, lifestyle habits, factors related to UV radiation, and reproductive and sexual health characteristics. In the codominant model, the TT genotype of rs2280883 was associated with slightly higher odds of skin tumors compared with the CC genotype (penalized OR = 1.02), whereas the TC genotype showed no difference in odds (penalized OR = 1.00). In the dominant model, carriers of the T allele (TT + CT) had lower odds of skin tumors compared with CC homozygotes (penalized OR = 0.85). In the recessive model, the TT genotype was associated with higher odds compared with the CT + CC genotypes (penalized OR = 1.18), while no difference was observed in the overdominant model (penalized OR = 1.00). Age ≤54 years was associated with higher odds of skin tumors compared with age ≥55 years across all genetic model specifications, with penalized ORs ranging from 3.03 to 4.82. Overweight was associated with slightly higher odds in the dominant and recessive models (penalized OR = 1.03), whereas the penalized OR was 1.00 in the codominant and overdominant models. Previous sexually transmitted infection was associated with lower odds in the dominant and recessive models (penalized OR = 0.86), while the penalized OR was 1.00 in the codominant and overdominant models. The penalized ORs for obesity, marital status, employment status, and tanning bed use were 1.00 across the genetic model specifications. No variables were selected by LASSO regression for vulvar or ectocervical tumors (Supplementary Table S1). Similarly, neither the rs3761548 genotype under any genetic model nor the other evaluated variables were selected in the LASSO models for skin, vulvar, or ectocervical tumors (Supplementary Table S2). To further explore whether age was associated with differences in other characteristics that could potentially contribute to the observed findings, we additionally compared anthropometric status, marital status, employment status, history of sexually transmitted infections, and tanning bed use between patients aged ≤54 and ≥55 years separately within each tumor group. These additional findings suggested that age was associated with some demographic and behavioral characteristics within the tumor groups, particularly in the skin and vulvar tumor groups (Supplementary Table S3).

## 4. Discussion

Analysis of sociodemographic data showed that women with skin and vulvar tumors were significantly older than those with ectocervical tumors. According to the literature, advanced age is recognized as a risk factor for the development of invasive skin SCC [25].

Women with skin and vulvar tumors also had lower educational levels, higher unemployment rates, lower single marital status and a greater prevalence of obesity. In contrast, women with ectocervical tumors were younger had lower BMI and consumed alcohol more frequently.

When assessing UV exposure, the association with vulvar tumors was not expected, as this skin is in sun protected anatomical site. Most study participants (85–94%) reported substantial sun exposure. In our study, sunburns were frequently reported across all three groups, and no significant differences were observed between the groups. This finding contrasts with previous reports showing that cutaneous squamous cell tumors are associated with a history of sunburn, as well as with HPV infection [26,27].

Significant differences in tanning bed use were observed between the groups. This may be explained by the younger age of women with ectocervical tumors, who were more likely to use tanning beds, whereas women with skin and vulvar tumors were older and reported less frequent tanning bed use. As tanning beds were introduced in Europe during the 1970s [28], they were not available to many of the older participants in our study during their younger years. Nevertheless, changing lifestyle trends may have contributed to their subsequent use among women, despite recommendations to avoid tanning beds and the implementation of a ban on their use by individuals under 18 years of age in Serbia since 2019 [29]. Our findings suggest that age was associated with some demographic and behavioral characteristics within the tumor groups, particularly in the skin and vulvar tumor groups (Supplementary Table S3).

No significant differences were observed between the groups with regard to smoking, as approximately half of the women in each group were smokers. According to the literature, smoking is strongly associated with the development of skin and cervical squamous cell tumors [30,31].

Similarly, no significant differences were observed in oral contraceptive use among the study groups. Although the literature suggests that oral contraceptive use is associated with the development of skin and cervical squamous cell tumors, its use was uncommon across all groups in our study. However, oral contraceptive use was somewhat more frequent among women with ectocervical tumors [31,32].

Women with ectocervical tumors had an earlier age at menarche and were more likely to have had their first sexual intercourse before the age of 17 years, as well as a greater number of lifetime sexual partners, but did not have a higher parity than women with skin and vulvar tumors. A history of sexually transmitted infection was significantly more frequently reported in the group of women with ectocervical tumors (91%). This finding is expected, as sexually transmitted infections, particularly HPV infection, are the primary cause of ectocervical SCC, accounting for approximately 90% of cases. About 10% of infected individuals develop persistent HPV infection, and 1–4% of these individuals subsequently develop an invasive tumor. Immunosuppressive local microenvironment induced by Treg cells and *FOXP3* may promote HPV persistence and contribute to the development and progression of these lesions. However, the exact mechanism underlying the interaction between HPV and *FOXP3* is unknown yet [33]. The observed differences between the groups may therefore be partly attributable to differences in age and generation, which may reflect substantial changes in lifestyle and sexual behavior over time.

Recent studies have shown that, in some cancer types, the *FOXP3* gene is expressed in tumor cells themselves, where it is involved in important signaling pathways that may influence cancer development and progression [34].

Several studies have highlighted the role of genetic variants within the *FOXP3* gene, including rs3761548 and rs2280883, in modulating *FOXP3* expression and function, thereby affecting Treg activity and potentially disrupting immune homeostasis in inflammatory and autoimmune diseases [11,17,18].

The rs3761548 polymorphism is located at position −3279 in the promoter region of the *FOXP3* gene and may influence *FOXP3* transcription. Promoter variants are thought to affect the interaction of this regulatory region with RNA polymerase. According to the literature, the A allele is associated with decreased *FOXP3* transcription. Studies have shown that *FOXP3* binds to the conserved non-coding sequence 2 (CNS2) in a RUNX1- and CBF-β-dependent manner, contributing to the stability of Treg cells. Furthermore, CNS2 interacts specifically with the *FOXP3* promoter in Treg cells, thereby promoting stable *FOXP3* expression [35,36].

In our study, the frequency of the minor A allele of *FOXP3* rs3761548 was lower in the skin and vulvar tumor groups, but slightly higher in the ectocervical tumor group, compared with European reference populations. Allele frequencies did not differ significantly among the three groups (*p* = 0.066), nor did pairwise comparisons reveal significant differences after Bonferroni correction. For rs3761548, significant differences in genotype distribution among the groups were observed under all genetic models except the recessive model, mainly reflecting differences between the skin and ectocervical tumor groups. In univariable logistic regression analysis, the codominant, dominant, and overdominant models for rs3761548 were significantly associated with tumor location, with opposite associations observed for skin and ectocervical tumors. In the codominant model, the AC genotype was associated with lower odds of skin tumors but higher odds of ectocervical lesions. Similarly, the dominant and overdominant models were associated with lower odds of skin tumors and higher odds of ectocervical lesions. None of the genetic models for either of the two evaluated *FOXP3* SNPs was associated with vulvar tumors. However, after LASSO logistic regression analysis of the skin and ectocervical tumor groups, none of the genetic models for the *FOXP3* rs3761548 polymorphism remained statistically significant.

Literature data on the *FOXP3* rs3761548 gene polymorphism in squamous cell and other tumor types, particularly those affecting the skin, vulva, and uterine ectocervix, are heterogeneous and conflicting. Cezar-dos-Santos et al. reported that the homozygous rs3761548 AA genotype, which is associated with decreased *FOXP3* expression in women, was an independent predictor of protection against the progression of HSIL to cervical carcinoma [14]. The authors speculated that this genotype may exert a protective effect against cervical HPV infection.

In a recent study, Aranome et al. evaluated the association between the *FOXP3* rs3761548 polymorphism and sociodemographic, reproductive, and sexual behavior factors that may contribute to cervical cancer development [37]. They found that limited medical knowledge, low income, infrequent preventive examinations, smoking, and high parity were associated with an increased risk of cervical carcinoma. However, no significant association was observed between the rs3761548 polymorphism and cervical cancer risk.

Ivković et al. investigated the clinical relevance of *FOXP3* expression and the rs3761548 polymorphism in HPV-negative oral squamous cell carcinoma [38]. They observed stage-specific differences in *FOXP3* expression in tumor tissue; however, no significant association was found between *FOXP3* expression and the rs3761548 genotype. More precisely, genetic variants of this polymorphism showed no statistically significant association with sex, age, smoking, alcohol consumption, or nodal tumor status.

A more recent meta-analysis aimed to re-evaluate the association between the rs3761548 polymorphism and cancer risk [39]. The authors used two statistical approaches, considering sex-related genotypic differences and the statistical challenges associated with X-chromosome analysis. The results indicated that the rs3761548 A allele was associated with an increased risk of cancer in both White and Asian populations. In addition, the A allele and AA genotype were associated with an increased risk of breast cancer among Asian populations, whereas the AC genotype appeared to have a protective effect against acute lymphoblastic leukemia in White populations, potentially related to non-random X-chromosome inactivation.

They emphasize that the *FOXP3* gene could act as an oncogene or a tumor suppressor depending on ethnicity, cancer type, and genotype distribution among the studied groups. Furthermore, they proposed that *FOXP3* expression may be influenced by epigenetic modifications associated with the rs3761548 C allele.

There is data in the literature on the importance of haplotypes involving two *FOXP3* promoter polymorphisms, rs3761548 and rs2232365, particularly in breast cancer [40]. In cervical cancer precursor lesions, haplotype analysis revealed no significant associations, whereas rs2232365 was independently associated with HPV infection [14]. A similar analysis of our sample could be performed in a subsequent stage of the research to provide a more complete picture of *FOXP3* promoter variants in skin, vulvar, and ectocervical squamous cell tumors in our cohort.

The rs2280883 polymorphism is located in intron 9 (IVS9+459) of the *FOXP3* gene. Its location near a conserved transcriptional region may influence downstream splicing, resulting in reduced gene expression [19].

In our study, the frequency of the C allele of rs2280883 was lower in the skin and ectocervical tumor groups but higher in the vulvar tumor group compared with European reference populations. A statistically significant difference in allelic distribution was observed among the three groups, while pairwise analysis revealed a significant difference only between the skin and ectocervical tumor groups, which remained statistically significant after Bonferroni correction. No significant differences were observed between the skin and vulvar tumor groups or between the ectocervical and vulvar tumor groups. Comparison of the three tumor groups revealed significant differences in rs2280883 genotype distributions under all genetic models, again predominantly reflecting differences between the skin and ectocervical tumor groups. In univariable logistic regression analysis, the dominant, recessive, and overdominant models of rs2280883 were significantly associated with skin tumors. The dominant and overdominant models showed protective associations, whereas the recessive model was associated with higher odds. For ectocervical tumors, the dominant model was associated with higher odds, whereas the recessive model was associated with lower odds. None of the genetic models for either of the two evaluated *FOXP3* gene SNPs were significantly associated with vulvar tumors. After LASSO logistic regression analysis, the dominant and recessive models of rs2280883 remained significantly associated with lower and higher odds of skin tumors, respectively. After reviewing the literature, no studies were identified that examined the association between the rs2280883 polymorphism and skin or cervical tumors.

In the meta-analysis mentioned above, no significant association was found between the rs2280883 polymorphism and cancer susceptibility in the overall population [19]. However, subgroup analysis according to the genotyping method revealed a lower cancer risk among studies using polymerase chain reaction–restriction fragment length polymorphism (PCR-RFLP), under the allelic model (C vs. T), heterozygote model (TC vs. TT), and dominant model (CC + TC vs. TT). In the subgroup analysis by cancer type, the C allele and TC genotype were associated with a protective effect against all cancer types except breast cancer under these three genetic models (C vs. T, TC vs. TT, and CC + TC vs. TT).

In order to understand the complex mechanisms of carcinogenesis in squamous cell tumors, as well as cancer development, prognosis, and therapeutic options, *FOXP3* expression has been extensively studied, both independently and in conjunction with genetic markers.

Sun et al. observed an increased number of Treg cells with high *FOXP3* expression in the peripheral circulation of patients with head and neck squamous cell carcinoma and found that their presence was associated with tumor progression [41]. Immune evasion mediated by *FOXP3*-expressing Treg cells and programmed cell death regulation (PD-L1) axis has been best characterized in invasive neoplasms, but it may also play a role in the progression of premalignant lesions [42].

## 5. Conclusions

Our findings highlight the importance of understanding the interplay between genetic, environmental, and behavioral factors in the prevention and effective treatment of squamous cell tumors.

Further studies are warranted to better understand the cellular context and to develop stage-specific immune interventions for squamous cell tumors.

Our study found that distribution of the *FOXP3* rs2280883 polymorphism differed according to tumor location. It was significantly associated with cutaneous squamous cell tumors under both dominant (CC + CT vs. TT) and recessive (CC vs. TT + CT) genetic models. However, our study has a limitation regarding the relatively small number of samples analyzed in all three tumor groups. To confirm our conclusions, it would be advisable to expand the study cohorts.

Given the limited number of studies examining the role of *FOXP3* in sun-exposed skin, sun-protected vulvar skin, and the ectocervix, further investigation in larger-scale studies is encouraged to strengthen and validate our findings.

## Figures and Tables

**Table 1:** Sociodemographic data, anthropometric parameter, lifestyle habits, factors related to UV radiation, and reproductive and sexual health characteristics of women with skin, vulvar, and ectocervical tumors.

Characteristic	Skin	Vulva	Ectocervix	*p* ^a^	*p* ^b^	*p* ^c^	*p* ^d^
	n = 49	n = 33	n = 53				
Age (years), n (%)							
≤54	4 (8.2)	7 (21.2)	46 (86.8)	**<0.001**	0.108	**<0.001**	**<0.001**
≥55	45 (91.8)	26 (78.8)	7 (13.2)				
Anthropometric parameter, n (%)							
Normal or underweight (BMI ≤ 24.9 kg/m^2^)	20 (40.8)	16 (48.5)	34 (64.2)	**0.005**	**0.016**	**0.022**	0.148
Overweight (BMI = 25.0–29.9 kg/m^2^)	25 (51.0)	8 (24.2)	13 (24.5)				
Obesity (BMI ≥ 30.0 kg/m^2^)	4 (8.2)	9 (27.3)	6 (11.3)				
Educational level, n (%)							
Primary or no education	12 (24.5)	10 (30.3)	0 (0.0)	**<0.001**	0.349	**0.001**	**<0.001**
Secondary	21 (42.9)	17 (51.5)	31 (58.5)				
Tertiary or higher	16 (32.7)	6 (18.2)	22 (41.5)				
Marital status, n (%)							
Single	3 (6.1)	1 (3.0)	9 (17.0)	**<0.001**	0.165	**<0.001**	**0.007**
Widowed or divorced	28 (57.1)	13 (39.4)	7 (13.2)				
Married or cohabiting/in relationship	18 (36.7)	19 (57.6)	37 (69.8)				
Employment status, n (%)							
Employed	8 (16.3)	6 (18.2)	40 (75.5)	**<0.001**	1.000	**<0.001**	**<0.001**
Unemployed	41 (83.7)	27 (81.8)	13 (24.5)				
Smoking status, n (%)							
Smoker	27 (55.1)	16 (48.5)	28 (52.8)	0.840	0.556	0.818	0.695
Non-smoker	22 (44.9)	17 (51.5)	25 (47.2)				
Alcohol consumption, n (%)							
Yes	25 (51.0)	9 (27.3)	38 (71.7)	**<0.001**	**0.032**	**0.032**	**<0.001**
No	24 (49.0)	24 (72.7)	15 (28.3)				
Sun exposure, n (%)							
Yes	46 (93.9)	28 (84.8)	45 (84.9)	0.299	0.258	0.144	1.000
No	3 (6.1)	5 (15.2)	8 (15.1)				
Tanning beds, n (%)							
Yes	4 (8.2)	4 (12.1)	32 (60.4)	**<0.001**	0.708	**<0.001**	**<0.001**
No	45 (91.8)	29 (87.9)	21 (39.6)				
Sunburns, n (%)							
Yes	46 (93.9)	25 (75.8)	46 (86.8)	0.061	**0.024**	0.323	0.190
No	3 (6.1)	8 (24.2)	7 (13.2)				
Age at menarche (years), n (%)							
<13	7 (14.3)	6 (18.2)	22 (41.5)	**0.004**	0.636	**0.002**	**0.025**
≥13	42 (85.7)	27 (81.8)	31 (58.5)				
Age at first sexual intercourse (years), n (%)							
≤17	12 (24.5)	15 (45.5)	25 (47.2)	**0.040**	**0.048**	**0.017**	0.877
≥18	37 (75.5)	18 (54.5)	28 (52.8)				
Number of sexual partners during lifetime							
≤3	34 (69.4)	22 (66.7)	19 (35.8)	**0.001**	0.795	**0.001**	**0.005**
≥4	15 (30.6)	11 (33.3)	34 (64.2)				
Sexual partner during last 6 months, n (%)							
0	41 (83.7)	24 (72.7)	8 (15.1)	**<0.001**	0.231	**<0.001**	**<0.001**
≥1	8 (16.3)	9 (27.3)	45 (84.9)				
Contraception, n (%)							
Yes	12 (24.5)	8 (24.2)	23 (43.4)	0.069	0.980	**0.044**	0.072
No	37 (75.5)	25 (75.8)	30 (56.6)				
Previous sexually transmitted infection, n (%)							
Yes	30 (61.2)	26 (78.8)	48 (90.6)	**0.002**	0.094	**<0.001**	0.199
No	19 (38.8)	7 (21.2)	5 (9.4)				
Parity, n (%)							
0	4 (8.2)	6 (18.2)	17 (32.1)	**0.002**	**0.043**	**<0.001**	0.276
1–2	44 (89.8)	23 (69.7)	28 (52.8)				
≥3	1 (2.0)	4 (12.1)	8 (15.1)				
Abortions, n (%)							
Yes	27 (55.1)	18 (54.4)	24 (46.2)	0.615	1.000	0.369	0.451
No	22 (44.9)	28 (53.8)	15 (45.5)				

Statistically significant *p*-values are shown in bold, as determined by the chi-square test or Fisher’s exact test, as appropriate. ^a^ Skin vs. vulvar vs. ectocervical tumors. ^b^ Skin vs. vulvar tumors. ^c^ Skin vs. ectocervical tumors. ^d^ Vulvar vs. ectocervical tumors.

**Table 2:** Distribution of *FOXP3* genotypes and alleles according to tumor location.

Characteristic	Skin	Vulva	Ectocervix	*p* ^a^	*p* ^b^	*p* ^c^	*p* ^d^
	n = 49	n = 33	n = 53				
rs3761548							
Codominant model							
CC	25 (51.0)	11 (33.3)	12 (22.6)	**0.043**	0.229	**0.008**	0.544
AC	13 (26.5)	14 (42.4)	27 (50.9)				
AA	11 (22.5)	8 (24.2)	14 (26.4)				
Dominant model							
AA + AC	24 (49.0)	22 (66.7)	41 (77.4)	**0.011**	0.113	**0.003**	0.276
CC	25 (51.0)	11 (33.3)	12 (22.6)				
Recessive model							
AA	11 (22.4)	8 (24.2)	14 (26.4)	0.897	0.850	0.642	0.822
CC + AC	38 (77.6)	25 (75.8)	39 (73.6)				
Overdominant model							
AC	13 (26.5)	14 (42.4)	27 (50.9)	**0.040**	0.133	**0.012**	0.442
AA + CC	36 (73.5)	19 (57.6)	26 (49.1)				
Allele							
C	63 (64.3)	36 (54.6)	51 (48.1)	0.066	0.211	**0.020**	0.412
A	35 (35.7)	30 (45.4)	55 (51.9)				
rs2280883							
Codominant model							
CC	9 (18.4)	6 (18.2)	13 (24.5)	**0.038**	0.095	**0.011**	0.702
TC	13 (26.5)	16 (48.5)	26 (49.1)				
TT	27 (55.1)	11 (33.3)	14 (26.4)				
Dominant model							
CC + TC	22 (44.9)	22 (66.7)	39 (73.6)	**0.009**	0.053	**0.003**	0.492
TT	27 (55.1)	11 (33.3)	14 (26.4)				
Recessive model							
CC	27 (55.1)	11 (33.3)	14 (26.4)	**0.009**	0.053	**0.003**	0.492
TT + TC	22 (44.9)	22 (66.7)	39 (73.6)				
Overdominant model							
TC	13 (26.5)	16 (48.5)	26 (49.1)	**0.040**	**0.041**	**0.019**	0.959
TT + CC	36 (73.5)	17 (51.5)	27 (50.9)				
Allele							
C	31 (31.6)	25 (37.9)	52 (49.1)	**0.037**	0.408	**0.011**	0.152
T	67 (68.4)	41 (62.1)	54 (50.9)				

Statistically significant *p*-values are shown in bold, as determined by the chi-square test or Fisher’s exact test, as appropriate. ^a^ Skin vs. vulvar vs. ectocervical tumors. ^b^ Skin vs. vulvar tumors. ^c^ Skin vs. ectocervical tumors. ^d^ Vulvar vs. ectocervical tumors.

**Table 3:** Univariable logistic regression analysis of *FOXP3* polymorphisms according to tumor location.

Models	Skin			Vulva			Ectocervix		
	OR	95%CI OR	*p*	OR	95%CI OR	*p*	OR	95%CI OR	*p*
rs3761548									
Codominant model									
CC	Ref.			Ref.			Ref.		
AC	0.292	0.13–0.68	**0.004**	1.177	0.48–2.92	0.724	3.000	1.29–6.97	**0.011**
AA	0.460	0.18–1.15	0.098	1.076	0.38–3.05	0.890	2.211	0.85–5.72	0.102
Dominant model									
CC	Ref.			Ref.			Ref.		
AA + AC	0.350	0.17–0.73	**0.005**	1.138	0.50–2.61	0.759	2.674	1.23–5.82	**0.013**
Recessive model									
CC + AC	Ref.			Ref.			Ref.		
AA	0.842	0.37–1.93	0.684	0.986	0.40–2.46	0.975	1.190	0.54–2.64	0.669
Overdominant model									
AA + CC	Ref.			Ref.			Ref.		
AC	0.396	0.19–0.85	**0.017**	1.142	0.52–2.53	0.744	2.115	1.04–4.30	**0.038**
rs2280883									
Codominant model									
CC	Ref.			Ref.			Ref.		
TC	0.653	0.24–1.79	0.408	1.504	0.51–4.40	0.456	1.034	0.42–2.58	0.942
TT	2.280	0.87–5.97	0.093	0.984	0.32–3.02	0.977	0.425	0.16–1.11	0.082
Dominant model									
TT	Ref.			Ref.			Ref.		
CC + TC	0.334	0.16–0.69	**0.003**	1.344	0.59–3.07	0.482	2.406	1.14–5.09	**0.022**
Recessive model									
TT + CT	Ref.			Ref.			Ref.		
CC	2.995	1.44–6.22	**0.003**	0.744	0.33–1.70	0.482	0.416	0.20–0.88	**0.022**
Overdominant model									
TT + CC	Ref.			Ref.			Ref.		
CT	0.378	0.18–0.81	**0.012**	1.520	0.69–3.35	0.299	1.760	0.87–3.56	0.115

**Table 4:** LASSO logistic regression results and penalized odds ratios for skin tumors.

Model	rs2280883							
	Codominant Model TC vs. CC TT vs. CC		Dominant Model TT + CT vs. CC		Recessive Model TT vs. CT + CC		Overdominant TT + CC vs. CT	
	β for λ_1se_	Penalized OR	β for λ_1se_	Penalized OR	β for λ_1se_	Penalized OR	β for λ_1se_	Penalized OR
Genetic model	00.018	1.00 1.02	−0.166	0.85	0.166	1.18	0	1.00
Age (≤54 vs. ≥55)	1.446	4.25	1.573	4.82	1.573	4.82	1.110	3.03
Anthropometric parameter								
Overweight vs. normal or underweight	0	1.00	0.028	1.03	0.028	1.03	0	1.00
Obesity vs. normal or underweight	0	1.00	0	1.00	0	1.00	0	1.00
Marital status								
Widowed or divorced vs. single	0	1.00	0.016	1.02	0.016	1.02	0	1.00
Married or cohabiting/in relationship vs. single	0	1.00	0	1.00	0	1.00	0	1.00
Employment status (employed vs. unemployed)	0	1.00	0	1.00	0	1.00	0	1.00
Previously sexually transmitted infection (yes vs. no)	0	1.00	−0.148	0.86	−0.148	0.86	0	1.00
Tanning bed (yes vs. no)	0	1.00	0	1.00	0	1.00	0	1.00

## Data Availability

The data presented in this study are available from the corresponding author upon reasonable request.

## Supplementary Materials

The following supporting information can be downloaded at: https://www.mdpi.com/article/10.3390/medicina62102000/s1, Supplementary Table S1. LASSO logistic regression results and penalized odds ratios for the *FOXP3* rs2280883 genotype in skin, vulvar, and ectocervical tumors. Supplementary Table S2. LASSO logistic regression results and penalized odds ratios for the *FOXP3* rs3761548 genotype in skin, vulvar, and ectocervical tumors. Supplementary Table S3. Comparison of age groups according to sociodemographic data, anthropometric parameters and factors related to UV radiation in skin, vulvar and ectocervical tumors.

## Abbreviations

The following abbreviations are used in this manuscript:

SCC	squamous cell carcinoma
UV	ultraviolet
HPV	human papillomavirus
AK	actinic keratosis
VIN	vulvar intraepithelial neoplasia
LSIL	low-grade squamous intraepithelial lesion
HSIL	high-grade squamous intraepithelial lesion
DVIN	differentiated type VIN
*FOXP3*	forkhead box P3 transcription factor
Tregs	regulatory T cells
SNP	single nucleotide polymorphism
UCCS	University Clinical Center of Serbia
FMUB	Faculty of Medicine, University of Belgrade
BMI	body mass index
HWE	Hardy–Weinberg equilibrium
MAF	minor allele frequency
