# Supplementary material for: Association of FOXP3 Gene Polymorphisms with Skin, Vulvar and Ectocervical Squamous Cell Tumors
Source: Medicina (Kaunas). 2026 Oct 15;62(10):2000. doi: 10.3390/medicina62102000 (PMC13707306; doi:10.3390/medicina62102000)
Supplement: Supplementary file 1 [file medicina-62-02000-s001.zip › medicina-4581466-supplementary.pdf]

Supplementary Table S1. LASSO logistic regression results and penalized odds ratios for the *FOXP3* rs2280883 genotype in skin, vulvar, and ectocervical tumors

**Model**	**rs2280883**							
	**Codominant model TC vs CC TT vs CC**		**Dominant model TT+CT vs CC**		**Recessive model TT vs CT+CC**		**Overdominant TT+CC vs CT**	
	**β (LASSO) for λ_{1se}**	**Penalized OR**	**β (LASSO) for λ_{1se}**	**Penalized OR**	**β (LASSO) for λ_{1se}**	**Penalized OR**	**β (LASSO) for λ_{1se}**	**Penalized OR**
SKIN								
**Genetic model**	0<br>0.018	1.00<br>1.02	-0.166	0.85	0.166	1.18	0	1.00
Age (≤ 54 vs ≥ 55)	1.446	4.25	1.573	4.82	1.573	4.82	1.110	3.03
Anthropometric parameter								
Overweight vs normal or underweight	0	1.00	0.028	1.03	0.028	1.03	0	1.00
Obesity vs normal or underweight	0	1.00	0	1.00	0	1.00	0	1.00
Marital status								
Widowed or divorced vs single	0	1.00	0.016	1.02	0.016	1.02	0	1.00
Married or cohabiting/in relationship vs single	0	1.00	0	1.00	0	1.00	0	1.00
Employment status (employed vs unemployed)	0	1.00	0	1.00	0	1.00	0	1.00
Previously sexually transmitted infection (yes vs no)	0	1.00	-0.148	0.86	-0.148	0.86	0	1.00
Tanning bed (yes vs no)	0	1.00	0	1.00	0	1.00	0	1.00

VULVA								
**Genetic model**	0<br>0	1.00<br>1.00	0<br>0	1.00<br>1.00	0<br>0	1.00<br>1.00	0<br>0	1.00<br>1.00
Age (≤ 54 vs ≥ 55)	0	1.00	0	1.00	0	1.00	0	1.00
Anthropometric parameter								
Overweight vs normal or underweight	0	1.00	0	1.00	0	1.00	0	1.00
Obesity vs normal or underweight	0	1.00	0	1.00	0	1.00	0	1.00
Marital status								
Widowed or divorced vs single	0	1.00	0	1.00	0	1.00	0	1.00
Married or cohabiting/in relationship vs single	0	1.00	0	1.00	0	1.00	0	1.00
Employment status (employed vs unemployed)	0	1.00	0	1.00	0	1.00	0	1.00
Previously sexually transmitted infection (yes vs no)	0	1.00	0	1.00	0	1.00	0	1.00
Tanning bed (yes vs no)	0	1.00	0	1.00	0	1.00	0	1.00
ECTOCERVIX								
**Genetic model**	0<br>0	1.00<br>1.00	0	1.00	0	1.00	0	1.00
Age (≤ 54 vs ≥ 55)	-1.652	0.19	-1.653	0.19	-1.653	0.19	-1.653	0.19
Anthropometric parameter								

Overweight vs normal or underweight	0	1.00	0	1.00	0	1.00	0	1.00
Obesity vs normal or underweight	0	1.00	0	1.00	0	1.00	0	1.00
Marital status								
Widowed or divorced vs single	0	1.00	0	1.00	0	1.00	0	1.00
Married or cohabiting/in relationship vs single	0	1.00	0	1.00	0	1.00	0	1.00
Employment status (employed vs unemployed)	0	1.00	0	1.00	0	1.00	0	1.00
Previously sexually transmitted infection (yes vs no)	0	1.00	0	1.00	0	1.00	0	1.00
Tanning bed (yes vs no)	0.070	1.07	0.070	1.07	0.070	1.07	0.070	1.07
Age at menarche (≥13 vs <13 years)	0	1.00	0	1.00	0	1.00	0	1.00
Age at first sexual intercourse (≥18 vs ≤17 years)	0	1.00	0	1.00	0	1.00	0	1.00
Number of sexual partners during lifetime (≥4 vs ≤3)	0	1.00	0	1.00	0	1.00	0	1.00
Sexual partner during last 6 months (yes vs no)	0.612	1.84	0.612	1.84	0.612	1.84	0.612	1.84
Contraception (yes vs no)	0	1.00	0	1.00	0	1.00	0	1.00

Supplementary Table S2. LASSO logistic regression results and penalized odds ratios for the *FOXP3* rs3761548 genotype in skin, vulvar, and ectocervical tumors

**Model**	**rs3761548**							
	**Codominant model AC vs CC AA vs CC**		**Dominant model AA+AC vs CC**		**Recessive model AA vs CC+AC**		**Overdominant AC vs AA + CC**	
	**β (LASSO) for λ_{1se}**	**Penalized OR**	**β (LASSO) for λ_{1se}**	**Penalized OR**	**β (LASSO) for λ_{1se}**	**Penalized OR**	**β (LASSO) for λ_{1se}**	**Penalized OR**
SKIN								
**Genetic model**	0<br>0	1.00<br>1.00	0	1.00	0	1.00	0	1.00
Age (≤ 54 vs ≥ 55)	1.230	3.42	1.449	4.26	1.449	4.26	1.230	3.42
Anthropometric parameter								
Overweight vs normal or underweight	0	1.00	0	1.00	0	1.00	0	1.00
Obesity vs normal or underweight	0	1.00	0	1.00	0	1.00	0	1.00
Marital status								
Widowed or divorced vs single	0	1.00	0	1.00	0	1.00	0	1.00
Married or cohabiting/in relationship vs single	0	1.00	0	1.00	0	1.00	0	1.00
Employment status (employed vs unemployed)	0	1.00	0	1.00	0	1.00	0	1.00
Previously sexually transmitted infection (yes vs no)	0	1.00	0	1.00	0	1.00	0	1.00
Tanning bed (yes vs no)	0	1.00	0	1.00	0	1.00	0	1.00

VULVA								
**Genetic model**	0<br>0	1.00<br>1.00	0<br>0	1.00<br>1.00	0<br>0	1.00<br>1.00	0<br>0	1.00<br>1.00
Age (≤ 54 vs ≥ 55)	0	1.00	0	1.00	0	1.00	0	1.00
Anthropometric parameter								
Overweight vs normal or underweight	0	1.00	0	1.00	0	1.00	0	1.00
Obesity vs normal or underweight	0	1.00	0	1.00	0	1.00	0	1.00
Marital status								
Widowed or divorced vs single	0	1.00	0	1.00	0	1.00	0	1.00
Married or cohabiting/in relationship vs single	0	1.00	0	1.00	0	1.00	0	1.00
Employment status (employed vs unemployed)	0	1.00	0	1.00	0	1.00	0	1.00
Previously sexually transmitted infection (yes vs no)	0	1.00	0	1.00	0	1.00	0	1.00
Tanning bed (yes vs no)	0	1.00	0	1.00	0	1.00	0	1.00
ECTOCERVIX								
**Genetic model**	0<br>0	1.00<br>1.00	0	1.00	0	1.00	0	1.00
Age (≤ 54 vs ≥ 55)	-1.653	0.19	-1.653	0.19	-1.653	0.19	-1.653	0.19
Anthropometric parameter								

Overweight vs normal or underweight	0	1.00	0	1.00	0	1.00	0	1.00
Obesity vs normal or underweight	0	1.00	0	1.00	0	1.00	0	1.00
Marital status								
Widowed or divorced vs single	0	1.00	0	1.00	0	1.00	0	1.00
Married or cohabiting/in relationship vs single	0	1.00	0	1.00	0	1.00	0	1.00
Employment status (employed vs unemployed)	0	1.00	0	1.00	0	1.00	0	1.00
Previously sexually transmitted infection (yes vs no)	0	1.00	0	1.00	0	1.00	0	1.00
Tanning bed (yes vs no)	0.070	1.07	0.070	1.07	0.070	1.07	0.070	1.07
Age at menarche (<13 vs ≥13 years)	0	1.00	0	1.00	0	1.00	0	1.00
Age at first sexual intercourse (≤17 vs ≥18 years)	0	1.00	0	1.00	0	1.00	0	1.00
Number of sexual partners during lifetime (≤3 vs ≥4)	0	1.00	0	1.00	0	1.00	0	1.00
Sexual partner during last 6 months (yes vs no)	0.612	1.84	0.612	1.84	0.612	1.84	0.612	1.84
Contraception (yes vs no)	0	1.00	0	1.00	0	1.00	0	1.00

Supplementary Table S3. Comparison of age groups according to sociodemographic data, anthropometric parameters and factors related to UV radiation in skin, vulvar and ectocervical tumors

Characteristic	SKIN		p*	VULVA		p*	ECTOCERVIX		p*
	≤54<br>n = 4	≥55<br>n = 45		≤54<br>n = 46	≥55<br>n = 7		≤54<br>n = 7	≥55<br>n = 26	
Anthropometric parameter									
Normal or underweight	3 (75.0)	17 (37.8)	0.514	32 (69.6)	2 (28.6)	0.065	4 (57.1)	12 (46.2)	0.868
Overweight	1 (25.0)	24 (53.3)		9 (19.6)	4 (57.1)		1 (14.3)	7 (26.9)	
Obesity	0 (0.0)	4 (8.9)		5 (10.9)	1 (14.3)		2 (28.6)	7 (26.9)	
Marital status									
Single	0 (0.0)	3 (6.7)	**0.041**	9 (19.6)	0 (0.0)	**0.044**	1 (14.3)	0 (0.0)	0.096
Single, widowed or divorced	0 (0.0)	28 (62.2)		4 (8.7)	3 (42.9)		1 (14.3)	12 (46.2)	
Married or cohabiting/in relationship	4 (100.0)	14 (31.1)		33 (71.7)	4 (57.1)		5 (71.4)	14 (53.8)	
Employment status									
Unemployed	0 (0.0)	41 (91.1)	**< 0.001**	7 (15.2)	6 (85.7)	**< 0.001**	5 (71.4)	22 (84.6)	0.584
Employed	4 (100.0)	4 (8.9)		39 (84.8)	1 (14.3)		2 (28.6)	4 (15.4)	
STDs									
Yes	1 (25.0)	18 (40.0)	1.000	42 (91.3)	6 (85.7)	0.522	6 (85.7)	20 (76.9)	1.000
No	3 (75.0)	27 (60.0)		4 (8.7)	1 (14.3)		1 (14.3)	6 (23.1)	
Tanning bed									
Yes	1 (25.0)	3 (6.7)	0.297	31 (67.4)	1 (14.3)	**0.012**	2 (28.6)	2 (7.7)	0.190
No	3 (75.0	42 (93.3)		15 (32.6)	6 (85.7)		5 (71.4)	24 (92.3)	

*Significance level of 0.05 according to Fisher's exact test.
